# A Multicenter, Single-Arm, Prospective Trial to Evaluate Efficacy and Safety of Dose-Dense Methotrexate, Vinblastine, Doxorubicin, and Carboplatin (DD-MVACarbo) Chemotherapy for Cisplatin-Ineligible Patients with Advanced Urothelial Cancer: Study Protocol of the CARBUNCLE Trial

**DOI:** 10.3390/mps7040058

**Published:** 2024-07-29

**Authors:** Makito Miyake, Satoshi Anai, Yusuke Iemura, Kazuki Ichikawa, Tatsuki Miyamoto, Atsushi Tomioka, Masaomi Kuwada, Yoshitaka Itami, Yukinari Hosokawa, Yoshiaki Matsumura, Eijiro Okajima, Kazumasa Torimoto, Nobutaka Nishimura, Mitsuru Tomizawa, Takuto Shimizu, Shunta Hori, Yosuke Morizawa, Daisuke Gotoh, Yasushi Nakai, Kiyohide Fujimoto

**Affiliations:** 1 Department of Urology, Nara Medical University, Kashihara 634-8522, Nara, Japan; nobunishimura11@gmail.com (N.N.); tomimit.com@gmail.com (M.T.); takutea19@gmail.com (T.S.); horimaus@gmail.com (S.H.); tigers.yosuke@gmail.com (Y.M.); dgotou@gmail.com (D.G.); nakaiyasusiuro@live.jp (Y.N.); kiyokun@naramed-u.ac.jp (K.F.); 2 Department of Urology, Nara Prefectural Seiwa Medical Center, Ikoma 636-0802, Nara, Japan; jpjpg.jbrk@gmail.com; 3 Department of Urology, Yamatotakada Municipal Hospital, Yamatotakada 635-8501, Nara, Japan; housevillagenmu@yahoo.co.jp (Y.I.); aburatani40@gmail.com (K.I.); 4 Department of Urology, Takai Hospital, Tenri 632-0006, Nara, Japan; tatsuki8770@gmail.com; 5 Department of Urology, Saiseikai Chuwa Hospital, Sakurai 633-0054, Nara, Japan; tomioka515@yahoo.co.jp; 6 Department of Urology, Matsusaka Chuo General Hospital, Matsusaka 515-0818, Mie, Japan; masaomikuwada@gmail.com; 7 Department of Urology, Tane General Hospital, Osaka 550-0025, Osaka, Japan; y.itami.324@gmail.com (Y.I.); yukinari46@nyc.odn.ne.jp (Y.H.); 8 Department of Urology, Nara City Hospital, Nara 630-8305, Nara, Japan; ymatsu0825@gmail.com (Y.M.); e-okajima@nara-jadecom.jp (E.O.); 9 Department of Urology, Nara Prefecture General Medical Center, Nara 630-8054, Nara, Japan; torimoto@nara-hp.jp

**Keywords:** advanced urothelial carcinoma, systemic chemotherapy, combination chemotherapy, cisplatin eligibility, carboplatin

## Abstract

Unresectable, metastatic, advanced urothelial carcinoma (aUC) is an aggressive disease and is treated with platinum-containing first-line chemotherapy, followed by immune checkpoint inhibitors and antibody–drug conjugates. Response to first-line chemotherapy is a vital priority in sequential treatment strategies because a better response to first-line chemotherapy is associated with a better response to subsequent therapies. Gemcitabine plus carboplatin chemotherapy is conventionally recommended for cisplatin-ineligible patients. This multicenter, single-arm prospective trial will investigate whether dose-dense methotrexate, vinblastine, doxorubicin, and carboplatin (DD-MVACarbo) chemotherapy is superior to gemcitabine plus carboplatin chemotherapy in terms of efficacy in platinum-naïve, cisplatin-ineligible patients with aUC. After screening and registration, a total of 46 patients will be treated with this novel chemotherapy regimen. The primary endpoint is the objective response rate. The secondary endpoints include disease control rate, patient-reported outcomes, and adverse events. No evidence of this novel intervention is available as of July 2024. The results are expected to change the standard of care and improve the management of patients with aUC.

## 1. Introduction

Urothelial carcinoma (UC) originates from cells lining the upper urinary tract (renal pelvis and ureter), bladder, and urethra. According to Global Cancer Statistics 2020, bladder cancer is the seventh most prevalent cancer among males globally and the thirteenth most common cancer in Japan [1,2]. Unresectable, metastatic, advanced UC (aUC) is an aggressive disease that typically demonstrates a median overall survival of approximately 40 months when initially treated with platinum-containing first-line (1L) chemotherapy, followed by immune checkpoint inhibitors (ICIs), such as avelumab and pemubrolizumab, and antibody–drug conjugates, such as enfortumab vedotin (EV) [3,4].

Response to 1L chemotherapy is a vital priority in sequential treatment strategies because a better response to 1L chemotherapy is associated with a better response to the subsequent ICIs and drug conjugates [3,5,6]. Clinical practice guidelines from the European Association of Urology and National Comprehensive Cancer Network recommend gemcitabine plus cisplatin (GC) combination chemotherapy or dose-dense methotrexate, vinblastine, doxorubicin (adriamycin), and cisplatin (DD-MVAC) combination chemotherapy as a 1L systemic treatment setting for cisplatin-eligible patients with aUC [7,8]. However, 30–50% of patients with aUC are reported to be ineligible for cisplatin in real-world clinical practice [9]. Gemcitabine plus carboplatin (GCarbo) combination chemotherapy is recommended as an alternative regimen for cisplatin-ineligible and carboplatin-eligible patients. Dogliotti et al. conducted phase 2 randomized trials comparing the toxicity and oncological efficacy of GC and GCarbo combination chemotherapies [10]. Although no differences were noted in the overall toxicity profiles, overall response rates (ORRs) were 49.1% for GC combination chemotherapy (complete response (CR), 14.5%; partial response (PR), 34.5%) and 40.0% for GCarbo combination chemotherapy (CR, 1.8%; PR, 38.2%), and median time to progression and survival were 8.3 and 12.8 months for GC combination chemotherapy and 7.7 and 9.8 months for GCarbo combination chemotherapy, respectively. A meta-analysis of 286 aUC patients from four randomized trials concluded that cisplatin-based chemotherapy was more likely to achieve a CR and an objective response (*p* = 0.005 and 0.02, respectively) [11]. There is an urgent need to develop a novel chemotherapy regimen that is comparable to GC combination chemotherapy for cisplatin-ineligible patients in terms of the safety profile and oncological efficacy, including response and survival.

Recently, two randomized controlled trials, namely, the EV-302 trial and CheckMate-901 trial, have demonstrated that novel combination therapies used as the 1L setting outperformed the conventional 1L platinum-based chemotherapy [12,13]. EV plus pembrolizumab combination therapy significantly prolonged progression-free survival (PFS) and overall survival (OS) by reducing the risk of progression by 55% and the risk of death by 53%, respectively, as compared to the chemotherapy arm (GC or GCarbo) [12]. However, there is still a scarcity of effective therapies that can be used after EV plus pembrolizumab combination therapy. The CheckMate-901 trial showed that GC plus nivolumab combination therapy and GC alone improved the outcomes of patients with previously untreated aUC [13]. One of the major limitations is that there is no evidence regarding the clinical efficacy of conventional platinum-based chemotherapy plus nivolumab in cisplatin-ineligible patients.

The aim of this prospective, multicenter, single-arm trial is to investigate the efficacy and safety of DD-MVA and carboplatin (DD-MVACarbo) combination chemotherapy for cisplatin-ineligible patients with aUC.

## 2. Trial Design

**Figure 1:**
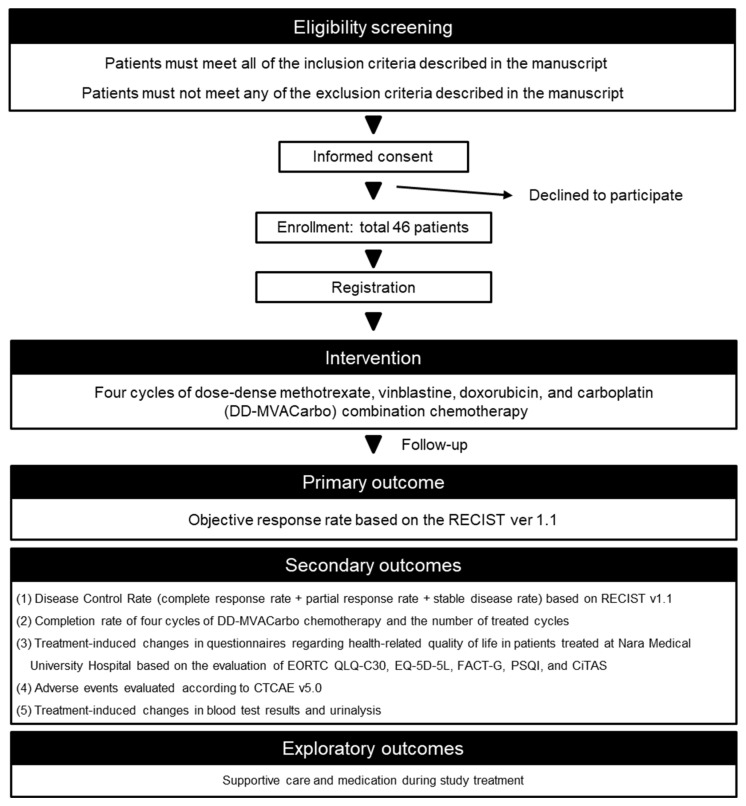
**Flow chart of the trial procedure.** RECIST, Response Evaluation Criteria in Solid Tumors; EORTC QLQ-C30, European Organization for Research and Treatment of Cancer Quality of Life Questionnaires-Core30; EQ-5D-5L, EuroQol 5-Dimension 5-Level; FACT-G, Functional Assessment of Cancer Therapy-General; PSQI, Pittsburgh Sleep Quality Index; CiTAS, chemotherapy-induced taste alteration scale; CTCAE, Common Terminology Criteria for Adverse Events.

This clinical trial is a prospective, multicenter, single-arm trial for aUC patients that is ongoing at eight medical institutes: Nara Medical University Hospital, Nara Prefectural Seiwa Medical Center, Yamatotakada Municipal Hospital, Takai Hospital, Saiseikai Chuwa Hospital, Matsusaka Chuo General Hospital, Tane General Hospital, and Nara City Hospital. Nara Prefecture General Medical Center will participate in the collaborative institute in July 2024. The flow chart of the trial procedure and outcomes are shown in Figure 1. The trial design and protocol adhere to the Recommendations for Interventional Trials (SPIRIT) criteria [14]. This trial complies with the Declaration of Helsinki for human investigations. The ethical clearance and final study protocol (version 1.1, 26 February 2024), including subject information, informed consent forms, and associated documents, were approved by the Certified Review Board of Nara Medical University (institution ID: CRB5200002). Informed and written consent forms will be mandatory before participating in the study. This clinical trial was prospectively registered with the Japan Registry of Clinical Trials on 7 May 2024 (reference number: jRCTs051240026). The URL for the trial registry record is available at https://jrct.niph.go.jp/latest-detail/jRCTs051240026 (accessed on 23 January 2024).

## 3. Procedure

### 3.1. Inclusion Criteria

Patients will undergo a general workup and a radiographic examination, including computed tomography (CT) or magnetic resonance imaging (MRI). Tumors were staged and graded according to the eighth edition of the American Joint Committee on Cancer TNM classification [15].

Prior to enrollment in this trial, patients must meet the following inclusion criteria:

- Patients with bladder, renal pelvic, ureteral, or urethral cancer;
- Patients with at least one evaluable target lesion according to Response Evaluation Criteria in Solid Tumors (RECIST) v1.1 (primary lesion or metastatic lesion); patients with cT1–4a N1–3 M0 bladder UC or cT1–3 N1–2 M0 upper urinary tract UC who will receive chemotherapy as a neoadjuvant setting are included;
- Age, 20–85 years;
- No treatment history of platinum-based chemotherapy (This trial does not exclude patients previously treated with non-platinum-based therapy.);
- Patients who meet at least one of the following cisplatin ineligibility criteria:
  - Glomerular filtration rate (GFR), 30–60 mL/min/1.73 m^2^;
  - Eastern Cooperative Oncology Group Performance Status (ECOG-PS), grade 2 or more;
  - Common Terminology Criteria for Adverse Events (CTCAE) version 5.0, hearing impaired, grade 2 or more;
  - CTCAE version 5.0, neuropathy, grade 2 or more;
  - New York Heart Association Class III or IV heart failure.
- Sufficient bone marrow and organ function within 30 days prior to study registration based on the following laboratory findings:
  - Hemoglobin, 9.0 g/dL or more;
  - White blood cell count, 3000–12,000/mm^3^;
  - Neutrophil count, ≥1500/mm^3^;
  - Platelet count, ≥100,000/mm^3^;
  - Aspartate aminotransferase, <100 IU/L and alanine aminotransferase, <100 IU/L.

### 3.2. Exclusion Criteria

Prior to enrollment in this trial, patients must not meet any of the following exclusion criteria:

- Patients who have difficulties with agreeing to participate in the trial on their own;
- Patients with contraindications for the anticancer drugs used in this trial;
- Patients deemed inappropriate for enrollment according to the physician’s judgment.

### 3.3. DD-MVACarbo Combination Chemotherapy

**Figure 2:**
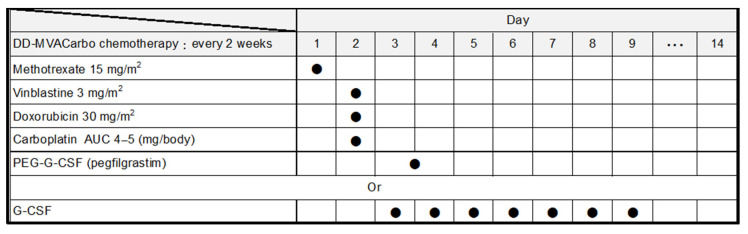
**Treatment regimen of DD-MVACarbo.** AUC, area under the curve; PEG, polyethylene glycol; G-CSF, granulocyte colony-stimulating factor. Filled circles indicate administration of the drug.

All anticancer drugs are administered intravenously. The chemotherapy schedule is 15 mg/m^2^ methotrexate on day 1, 3 mg/m^2^ vinblastine on day 2, 30 mg/m^2^ doxorubicin on day 2, and a target area under the curve (AUC) of 4–5 carboplatin on day 2, with support using 3.6 mg of subcutaneous pegfilgrastim on day 3 or 4, or alternatively standard granulocyte colony-stimulating factor from days 3 to 9 according to the neutrophil count, every 2 weeks for a total of four cycles (Figure 2). Although the dose of methotrexate is 30 mg/m^2^ in the chemotherapy schedule of DD-MVAC, dose reduction is recommended for patients with renal impairment [16]. Carboplatin doses were calculated using the Calvert formula [17], with a target AUC calculated using GFR via the Cockcroft–Gault equation [18] as follows: carboplatin dose (mg) = target AUC × [GFR (mL/min) + 25].

The VESPER trial compared the clinical efficacy and safety of six cycles of DD-MVAC with that of four courses of GC in the perioperative setting [19]. Only 127 (58%) out of 218 patients in the DD-MVAC group completed six cycles of DD-MVAC [20]. Additionally, The median cycle number of DD-MVAC was four (interquartile range, 4–4); >90% of patients completed all scheduled cycles in the real-world clinical practice [21]. Although those patients were cisplatin-eligible, targeted patients were cisplatin-ineligible in the present CARBUNCLE trial. Anticancer drugs could have a lower tolerability in cisplatin-eligible patients than that in cisplatin-eligible patients. Therefore, the planned cycles of DD-MVACarbo were set to a maximum of four in this trial.

### 3.4. Treatment Schedule and Data Collection

**Figure 3:**
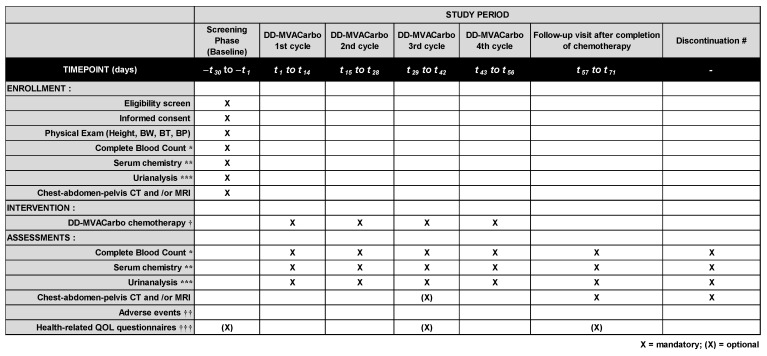
**Enrollment, intervention, and assessment schedule of the CARBUNCLE trial.** Blood exams and urinalysis will be performed on day 1 of each cycle of DD-MVACarbo, and radiologic exams will be performed after four cycles of DD-MVACarbo. Routine visits to the hospital are not specified on days 9–14 in this trial. However, the patients will be asked to visit the hospital for an examination if clinically indicated. Chest–abdomen–pelvis computed tomography (CT) and/or magnetic resonance imaging (MRI) should be performed for TNM classification and evaluation of tumor response. The case report forms (CRFs) will include information regarding past history, concomitant medications, and any medications taken after the treatment. BW, body weight; BT, body temperature; BP, blood pressure. * hemoglobin, red blood cell count, white blood cell count and fractions, and platelet count; ** aspartate transaminase (AST), alanine transaminase (ALT), total bilirubin, serum albumin, serum creatinine, and C-reactive protein (CRP); *** urinary protein semiquantified by urine dipstick test; † following cycles can be delayed at most 7 days due to adverse events; †† according to the Common Toxicity Criteria for Adverse Events (CTCAE v5.0) translated into Japanese; ††† EORTC QLQ-C30, EQ-5D-5L, FACT-G, PSQI, and CiTAS questionnaires for patients who will be treated at Nara Medical University Hospital; # data will be collected at the end of the trial for patients who discontinued chemotherapy without completion of the planned cycles.

Patient data will be collected before chemotherapy, during chemotherapy, and within 28 days of chemotherapy. Data will be collected at the end of the trial for patients who discontinued chemotherapy without completion of the planned cycles (Figure 3). Patients who will be treated at Nara Medical University Hospital will be asked to complete the following patient-reported outcome questionnaires: European Organization for Research and Treatment of Cancer Quality of Life Questionnaires-Core 30 (EORTC QLQ-C30), EuroQol 5-Dimension 5-Level (EQ-5D-5L), Functional Assessment of Cancer Therapy-General (FACT-G), Pittsburgh Sleep Quality Index (PSQI), and chemotherapy-induced taste alteration scale (CiTAS). All the collected data will be documented in specific case report forms (CRFs) for complete blood count, serum chemistry, CT/MRI, adverse events (AEs) using CTCAE v5.0, and tumor response to chemotherapy. AEs strongly related to the intervention of DD-MVACarbo chemotherapy are listed as follows: “Anemia”, “Febrile neutropenia”, “Constipation”, “Diarrhea”, “Fever”, “Neutropenia”, “Thrombocytoepenia”, “Anorexia”, “Dehydration”, “Tumor hemorrhage”, or “Tumor pain”. Any other potential AEs are recorded and grouped into “related AEs” or “unrelated AEs”. When serious AEs occur, investigators report to the Monitoring Committee.

To protect patients’ personal information, unique identification codes (CARBUNCLE IDs) will be assigned to all patients. All data and CRFs will be protected in password-accessible electronic data files on secure servers and lockable rooms, respectively, and only investigators will be able to access these files. All data and documents will be deleted and discarded five years after the trial ends, unless the data are being used for another study.

### 3.5. Sample Size Setting

The primary endpoint of this trial is the ORR (CR rate + PR rate) using RECIST v1.1. In Japan, GCarbo combination chemotherapy has been widely used in cisplatin-ineligible patients with aUC as of June 2024 [22]. Historical data from the Nara Urological Research and Treatment Group demonstrated that the ORR of patients who received 1L GCarbo combination chemotherapy was 41% [5]. Based on the results of a previous large-scale randomized controlled trial in which patients who received DD-MVAC chemotherapy had an ORR of 62% [23], we expected an equivalent ORR (62%) after treatment with DD-MVACarbo chemotherapy in this trial. We will investigate whether DD-MVACarbo chemotherapy (estimated ORR, 62%) is superior to GCarbo combination chemotherapy (actual ORR based on the historical data, 41%) using the ORR in this trial. Given that 15% of patients were ineligible for evaluation due to discontinuation of chemotherapy, withdrawal of consent, or serious deviation, a binomial test determined that the required sample size is at least 46 for trial cases to provide 80% power (β = 0.20) with an α level of 0.05 (two-sided).

### 3.6. Interim Analysis and Monitoring

We will not conduct an interim analysis of the clinical efficacy of DD-MVAC chemotherapy. However, the monitoring committee will independently evaluate whether the study is implemented in compliance with the study protocol and that the data are appropriately corrected according to a pre-arranged monitoring plan at the time when the first patients have been enrolled and once per year.

## 4. Expected Results and Statistical Analysis

The primary endpoint of this trial is the ORR. We expect that the lower limit of the 95% confidence interval (CI) of the ORR will be >41% (threshold response rate) in the FAS population, indicating that DD-MVACarbo chemotherapy is considered superior to conventional GCarbo chemotherapy. Detailed statistical analysis is shown below.

### 4.1. Statistical Analysis

Descriptive statistics will be computed for all the study variables. PRISM software version 10 (GraphPad Software, Inc., San Diego, CA, USA) will be used for statistical analyses and data plotting. A two-sided *p* value of less than 0.05 will be considered statistically significant.

The study endpoints are as follows:

- Primary outcome: ORR (CR rate + PR rate) based on RECIST v1.1;
- Secondary outcomes:
  (1) Disease Control Rate (sum of CR rate + PR rate + SD rate) based on RECIST v1.1;
  (2) Completion rate of four cycles of DD-MVACarbo chemotherapy and the number of treated cycles;
  (3) Treatment-induced changes in questionnaire responses regarding health-related quality of life in patients treated at Nara Medical University Hospital based on evaluations of EORTC QLQ-C30, EQ-5D-5L, FACT-G, PSQI, and CiTAS;
  (4) AEs evaluated according to CTCAE v5.0;
  (5) Treatment-induced changes in blood test results and urinalysis.
- Exploratory outcomes: supportive care and medication during study treatment.

The full analysis set (FAS) and per-protocol set populations will be analyzed separately for both the primary and secondary endpoints. The safety analysis set (SAS) is defined as the population of patients who are administered at least one anticancer drug in the DD-MVAC chemotherapy regimen. The SAS population will be analyzed to determine safety using the secondary endpoints.

If the lower limit of the 95% confidence interval (CI) of the ORR is >41% (threshold response rate) in the FAS population, then the DD-MVACarbo chemotherapy will be considered superior to conventional GCarbo chemotherapy. If the lower limit of the 95% CI of the ORR is ≤41%, then the DD-MVACarbo chemotherapy will not be considered superior to conventional chemotherapy. The amount of change in other postoperative observations over time will be analyzed using repeated measures analysis of variance, mixed-effects models for repeated measures, the Wilcoxon signed-rank sum test, or the Mann–Whitney U test for longitudinal data, as appropriate. A subgroup analysis will be performed to examine whether the effects of DD-MVACarbo chemotherapy differ from those of subgroups historically treated with GCarbo chemotherapy according to patient characteristics.

The amount of change in other postoperative observations over time will be analyzed using the Wilcoxon signed-rank sum test or the Mann–Whitney U test, as appropriate. The repeated measures analysis of variance and mixed-effects models for repeated measures will be applied to detect multiple change points in the longitudinal data, as previously described [24].

### 4.2. Dissemination

The results will be submitted to peer-reviewed journals for publication and presented at local and international conferences. The results will be made available to interested participants.

## 5. Discussion and Conclusion

aUC is an aggressive disease with a median overall survival of approximately 40 months when treated with platinum-containing first-line chemotherapy, followed by immune checkpoint inhibitors and antibody–drug conjugates. Response to first-line chemotherapy is a vital priority in sequential treatment strategies because a better response to 1L chemotherapy is associated with a better response to subsequent therapies. EV plus pembrolizumab combination therapy and GC plus nivolumab combination therapy outperformed conventional platinum-based chemotherapy as a 1L treatment setting for patients with aUC. One of the biggest concerns would be that not all the patients are eligible for EV plus pembrolizumab therapy as described in the ESMO Clinical Practice Guideline interim update on first-line therapy in advanced urothelial carcinoma [25]. Ineligibility criteria would include old age, autoimmune diseases, and poor performance status. We believe that DD-MVACarbo can be a useful alternative treatment modality for EV + pembrolizumab-ineligible patients with aUC.

There are still unmet medical needs regarding therapies that can be used after EV plus pembrolizumab combination therapy. Because our trial includes patients who did not receive any platinum-based chemotherapy previously, those who progress after this combination therapy can be treated with DD-MVACarbo as a second-line setting or later. Moreover, there is no evidence of the safety or efficacy of platinum-based chemotherapy plus nivolumab combination therapy in cisplatin-ineligible patients.

The goal of this trial is to determine the potential benefits of DD-MVACarbo chemotherapy in aUC patients. No data regarding this challenging intervention are available as of June 2024. The results are expected to change the standard of care and improve the management of aUC patients.

## Data Availability

The collected datasets used during this clinical trial are available from the corresponding author (M. Miyake) on reasonable request.
